# Vasorelaxant and Hypotensive Effects of Cheonwangbosimdan in SD and SHR Rats

**DOI:** 10.1155/2018/6128604

**Published:** 2018-07-10

**Authors:** Bumjung Kim, Cheolmin Jo, Ho-Young Choi, Kyungjin Lee

**Affiliations:** ^1^Department of Herbology, College of Korean Medicine, Kyung Hee University, Seoul 02447, Republic of Korea; ^2^Department of Herbology, Graduate School, Kyung Hee University, Seoul 02447, Republic of Korea

## Abstract

Historically, traditional herbal medicines (THMs) have been the conventional treatment strategy in the Korean medical system for treating many diseases. However, THMs have rarely been used to treat hypertension, and moreover few studies have investigated the interaction of blood pressure with the coadministration of synthetic antihypertensives. We aimed to evaluate the vasorelaxant and hypotensive effects of the traditional herbal prescription Cheonwangbosimdan (CWBSD; “Tianwangbuxindan” in Chinese) and the combination of CWBSD with amlodipine. CWBSD was extracted with distilled water at 100°C for 2 h. To investigate vasorelaxant activities, CWBSD with amlodipine (10 *μ*g/ml) was added cumulatively (10–1,000 *μ*g/ml) to isolated rat aortic rings precontracted using phenylephrine or potassium chloride in organ chambers. To investigate hypotensive effects, CWBSD (2,476 mg/kg) was orally administered with or without amlodipine (5 mg/kg) to spontaneously hypertensive rats (SHRs). CWBSD increased the relaxation of rat aortic rings induced by amlodipine (*P* < 0.01).* In vivo*, CWBSD coadministration with amlodipine also significantly decreased the blood pressure of SHRs compared to the amlodipine-treated group. These results suggested that CWBSD could be a useful herbal prescription to treat hypertension and we recommend establishing guidelines for the use of herbal medicines in conjunction with antihypertensive drugs, including amlodipine.

## 1. Introduction

Hypertension is a major risk factor for cardiovascular disease, chronic kidney disease, cerebrovascular disease, ischemic heart disease, stroke, and coronary heart disease. Approximately 30% of adults in the United States are not aware of their hypertension and in two-thirds of patients, hypertension is not being controlled [1]. According to the Korean National Health and Nutrition Examination Survey, the prevalence of hypertension among adults aged over 30 years was approximately 30% in Korea. The prevalence of hypertension has not been constant over time; the prevalence was 29% in 1998, decreased slightly to 24% in 2007, and then increased again to 28% in 2011 [2]. Despite the improvement in the treatment rate of hypertension, some older patients still have untreated and uncontrolled hypertension. Although the prevalence of hypertension has remained relatively stable over the last several decades, the number of older people with hypertension has been steadily increasing due to a rapidly aging society. In particular, isolated systolic hypertension, one of four subtypes of hypertension, is becoming more prevalent [3].

Historically, traditional herbal medicines (THMs) have been the conventional treatment method in the Korean medical system for treating various diseases. Traditional Korean medicine, which was discovered around 5,000 years ago, has peculiar medical theories and strategies to treat diseases [4]. In Korea, despite the long history of using THMs for the treatment of many diseases, they are rarely used in patients with hypertension due to a lack of evidence-based data. Moreover, few studies have been conducted on the interaction of synthetic antihypertensive drugs and THMs, even though hypertensive patients have been taking antihypertensive drugs such as amlodipine with traditional herbal prescriptions in Korea.

Over three-fourths of the world's population uses THMs, because they may be beneficial and have few side effects. However, some adverse effects and potential toxicities of THMs have been reported. Therefore, further study will be necessary to estimate their efficacy, quality, and safety [5]. Additionally, few studies have been conducted on the effect of coadministration of THMs and Western drugs such antihypertensive drugs on blood pressure.

THMs, composed of various components, may be beneficial to treat hypertension. Although patients with hypertension use THMs to improve their health, Western medical practitioners prohibit or discourage taking herbal medicine with antihypertensive drugs. Therefore, it is necessary to investigate the hypotensive activities of THMs and interactions of coadministration of amlodipine and herbal prescriptions. In our previous study, we investigated the vasorelaxant effects of 50 commonly used traditional herbal prescriptions on isolated rat aortic rings. Among them, Cheonwangbosimdan (CWBSD; “Tianwangbuxindan” in Chinese) showed the most remarkable vasorelaxant effects.

CWBSD is often used in Korea to treat depressive and anxiety disorders, including posttraumatic stress disorder and obsessive-compulsive disorder. This prescription is composed of 15 herbal medicines including Rehmanniae Radix, Coptidis Rhizoma, Acori Graminei Rhizoma, Ginseng Radix, Angelicae Gigantis Radix, Schisandrae Fructus, Asparagi Tuber, Liriopis seu Ophiopogonis Tuber, Thujae Semen, Zizyphi Semen, Scrophulariae Radix, Poria Sclerotium, Salviae Miltiorrhizae Radix, Platycodonis Radix, and Polygalae Radix. A previous study analyzed 7 bioactive compounds, including 5-hydroxymethyl-2-furaldehyde (5-HMF) from* Asparagus cochinchinensis*, berberine and coptisine from* Coptis japonica*, nodakenin from* Angelica gigas*, harpagoside and cinnamic acid from* Scrophularia buergeriana*, and *β*-asarone from* Acorus gramineus,* for quality control of CWBSD by HPLC-PDA [6]. In recent studies, CWBSD has been shown to have antioxidant [7], antidepressant [8], and neuroprotective effect [9], effects against Alzheimer's disease [10–12], effects that increase gamma-aminobutyric acid [13], and effects on the central nervous system and cardiovascular system [14].

To provide evidence and guidelines for the use of CWBSD or a combination of CWBSD and amlodipine in patients with hypertension, we investigated the vasorelaxant and hypotensive effects of CWBSD and the combination of CWBSD and amlodipine using isolated rat aortic rings and spontaneously hypertensive rats (SHRs).

## 2. Materials and Methods

### 2.1. Material and Extraction

The water extract of CWBSD was provided by the Korea Institute of Oriental Medicine in Korea. It is a complex powder extracted from 15 kinds of herbal medicines (Table 1). Extraction protocol was as follows: CWBSD was extracted once in distilled water at 100°C for 2 h using an electric extractor (COSMOS-660; Kyungseo Machine Co., Incheon, Korea). The extract was evaporated and freeze-dried to obtain a powder after filtration. The yield of CWBSD extract was 21.3%. The CWBSD powder was accurately weighed (0.1 g), suspended in 1 ml of distilled water, and placed into an ultrasonic device for 1 min for solubilization. The powder was completely dissolved in Krebs-Henseleit (KH) buffer and distilled water.

### 2.2. Animals

Sprague-Dawley rats (male, 240–260 g, 8 weeks old) and spontaneously hypertensive rats (SHR/lzm; male, 200–250 g; 8 weeks old) were purchased from Raonbio (Gyeonggi province, Korea) and housed under standard laboratory conditions (22 ± 2°C; lighting, 07:00–19:00) and were given food and water* ad libitum*. All procedures followed the animal welfare guidelines and were approved [KHUASP (SE)-16-143] by the Kyung Hee University Institutional Animal Care and Use Committee.

### 2.3. Preparation of Rat Aortic Rings

The rat thoracic aorta was isolated and stored in KH buffer (mM), NaCl 118.0, KCl 4.7, MgSO_4_ 1.2, KH_2_PO_4_ 1.2, CaCl_2_ 2.5, NaHCO_3_ 25.0, and glucose 11.1, and then oxygenated with a gas mixture of 95% O_2_–5% CO_2_. After careful removal of the connective tissue and surrounding fat, the aorta was cross-sectioned into 2 mm rings and placed in organ chambers containing 10 ml KH buffer at 37°C and pH 7.4. The rings were suspended between two tungsten stirrups for the measurement of isometric tension by a force transducer (Grass Instrument Co., USA). After equilibration under no tension for 30 min, the aortic rings were incubated for 1 h at a resting tension of 1.2 g. During the equilibration period, the KH buffer was changed every 15–20 min for 90 min. Isometric transducers connected to a data acquisition system (PowerLab, ADI instrument Co., Australia) were used to measure the changes in tension of the aortic rings.

### 2.4. Experimental Protocols

#### 2.4.1. Effects of CWBSD on Phenylephrine- (PE-) or Potassium Chloride- (KCl-) Induced Contraction

Studies were performed on rat aortic rings precontracted with PE (1 *μ*M) or KCl (60 mM) in KH buffer. Following equilibration period, the effect of various cumulative doses of CWBSD (10–1,000 *μ*g/ml) was measured. The vasorelaxant effect of CWBSD was expressed as a percentage relaxation of the response to PE- or KCl-induced contraction.

#### 2.4.2. Effects of CWBSD on Extracellular Ca^*2*+^-Induced Contraction via Receptor-Operative Ca^*2*+^ Channels (ROCCs) or Voltage-Dependent Ca^*2*+^ Channels (VDCCs)

The vasorelaxant effect of CWBSD (600 *μ*g/ml) was tested on ROCCs or VDCCs by PE (1 *μ*M) or KCl (60 mM) pretreatment. ROCCs are activated by PE and VDCCs are activated by KCl. After CWBSD preincubation for 10 min, endothelium-denuded aortic rings were treated with PE or KCl, and then the contractile response induced by CaCl_2_ (0.3–10 mM) was tested. The contraction responses induced by extracellular Ca^2+^ with or without (control) CWBSD preincubation were compared.

#### 2.4.3. Effects of CWBSD after Treatment of Amlodipine on PE-Induced Contraction

We investigated the concentration-dependent vasorelaxant activities of CWBSD (10–1,000 *μ*g/ml) after relaxing aortic rings with amlodipine (10 *μ*g/ml). The vasorelaxant effect of CWBSD was expressed as a percentage relaxation of the response to PE-induced contraction.

### 2.5. Measurement of Blood Pressure

The systolic blood pressure (SBP) of SHRs was measured using the noninvasive tail cuff system (CODA 8-Channel High Throughput Noninvasive Blood Pressure system, Kent Scientific Co. Ltd., Torrington, CT, USA). They were randomly divided into five groups of five animals each. CWBSD and amlodipine were dissolved in distilled water for this experiment. Each rat was orally given CWBSD (247.6 mg/kg), CWBSD (1,238 mg/kg), CWBSD (2,476 mg/kg), amlodipine (5 mg/kg), or coadministered amlodipine (5 mg/kg) and CWBSD (2,476 mg/kg). The blood pressure of SHRs was measured at 0 (control), 0.5, 1, 2, and 4 h after drug administration.

### 2.6. Statistical Analysis

Data are expressed as mean ± standard error of mean (SEM) and analyzed using IBM SPSS version 23.0 statistical analysis software (SPSS Inc., Chicago, IL, USA). Comparisons of analytical results were performed using Student's t-test or one-way analysis of variance (ANOVA) followed by Tukey's post hoc test. Data with p values less than 0.05 were considered statistically significant.

## 3. Results

### 3.1. Vasorelaxant Effects of CWBSD on PE- or KCl-Induced Contraction

**Figure 1:**
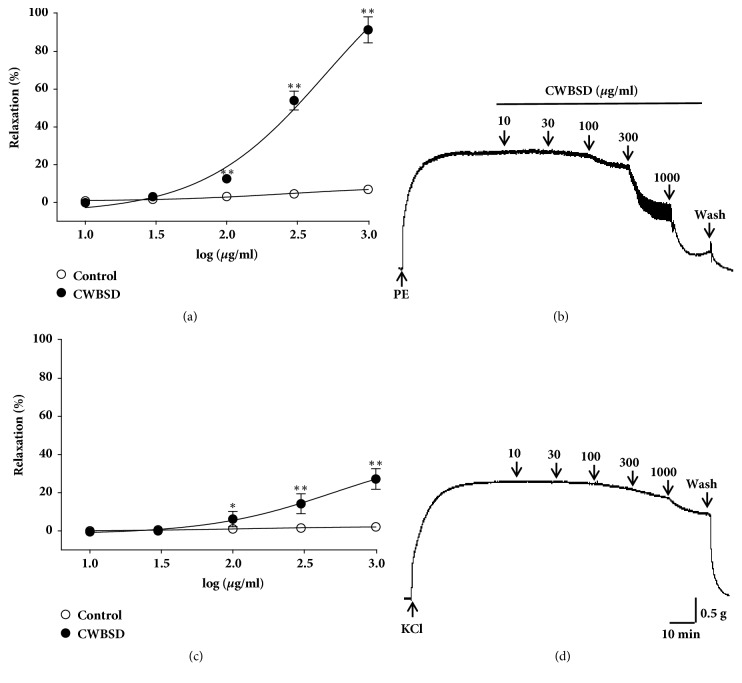
Vasorelaxant effects and representative traces of CWBSD treatment of PE- (1 *μ*M) ((a), (b)) or KCl- (60 mM) ((c), (d)) precontracted rat aortic rings. Values are expressed as mean ± SEM (n = 5–6). ^*∗*^*p* < 0.05 and ^*∗∗*^*p* < 0.01 vs. control.

CWBSD treatment led to a concentration-dependent vasorelaxant activity in endothelium-intact aortic rings precontracted with PE (1 *μ*M) or KCl (60 mM). The maximal relaxant effect was 91.5 ± 6.9% and 27.2 ± 5.4% at the concentration of 1,000 *μ*g/ml, respectively (Figure 1).

### 3.2. Vasorelaxant Effect of CWBSD on Extracellular ROCCs or VDCCs

**Figure 2:**
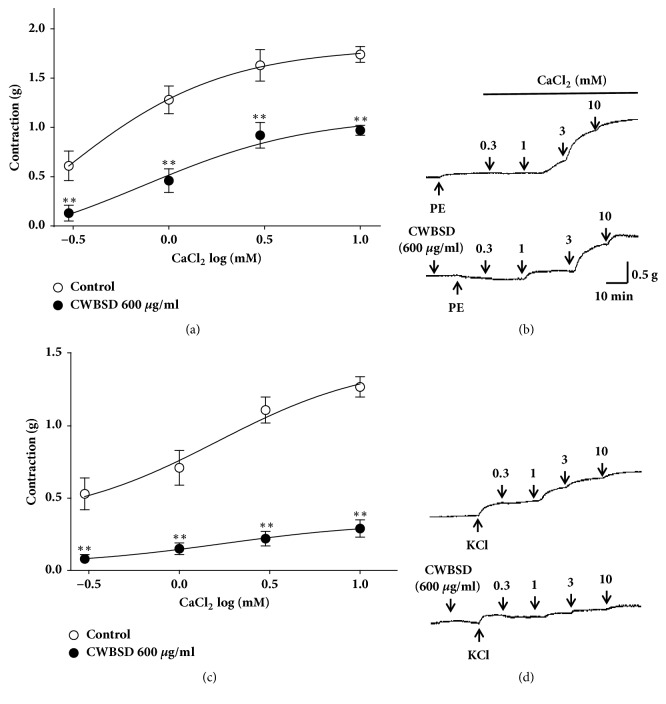
Inhibitory effects of CWBSD (600 *μ*g/ml) on the contraction induced by extracellular Ca^2+^ in endothelium-denuded rat aortic ring pretreated with PE (1 *μ*M) ((a), (b)) or KCl (60 mM) ((c), (d)) with or without (control) CWBSD. (b) and (d) show representative traces under the indicated conditions. Values are expressed as mean ± SEM (n = 5). ^*∗∗*^*P* < 0.01 vs. control.

Ca^2+^-induced contraction was obtained by cumulative addition of CaCl_2_ (0.3–10 mM). Pretreatment with CWBSD (600 *μ*g/ml) significantly inhibited the contraction induced by extracellular CaCl_2_ (10 mM) in Ca^2+^-free KH buffer and the contraction decreased to 0.97 ± 0.05 and 0.29 ± 0.06 compared to control group (1.74 ± 0.08 and 1.27 ± 0.07) in aortic rings pretreated with PE and KCl, respectively (Figure 2).

### 3.3. Vasorelaxant Effects of Coinjection of Amlodipine and CWBSD on PE-Induced Contraction

**Figure 3:**
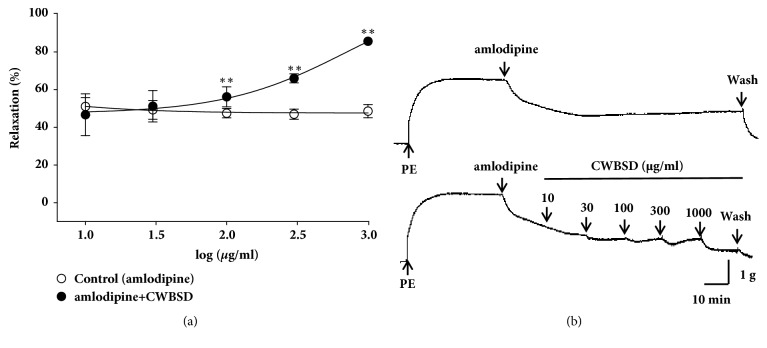
Vasorelaxant effects and representative traces of CWBSD after pretreatment of amlodipine (control) on PE- (1 *μ*M) ((a) and (b)) precontracted rat aortic rings. Values are expressed as mean ± SEM (n = 5–6). ^*∗∗*^*p* < 0.01 vs. control.

CWBSD more relaxed isolated rat aortic rings pretreatment with PE (1 *μ*M) which were relaxed by amlodipine (10 *μ*g/ml). The maximal relaxant effect of CWBSD (1,000 *μ*g/ml) was 85.7 ± 1.8% compared to control (amlodipine), 48.6 ± 3.5% (Figure 3).

### 3.4. Effects of CWBSD on Blood Pressure in SHRs

CWBSD (2,476 mg/kg) significantly decreased SBP to 172.7 ± 11.2 (*p *< 0.05) and 192.5 ± 6.8 (*p *< 0.05) at 1 h and 4 h after administration, respectively (Table 2).

### 3.5. Hypotensive Effects of Coadministration of Amlodipine and CWBSD in SHRs

Before oral administration of amlodipine or CWBSD (0 h), the SBP of the amlodipine-treated group was 218.8 ± 6.2 mmHg and that of the amlodipine and CWBSD combination treated group was 204.5 ± 12.0 mmHg. Four hours after the oral administration of amlodipine or the combination of amlodipine and CWBSD, the SBP of SHRs significantly decreased to 168.6 ± 1.5 mmHg (*p *< 0.01) or 138.2 ± 2.8 mmHg (*p *< 0.01) (Table 3), respectively. This effect was stronger in those treated with the combination of amlodipine and CWBSD treatment than those with amlodipine alone (*p *< 0.01) (Table 3).

## 4. Discussion

Recently, various studies have been conducted on the efficacy and safety of common traditional herbal prescriptions, which are widely used in Korea [15–20]. Their interactions with Western medicines also have been researched [21–23].

Hypertension is the most common chronic disease and the worldwide prevalence of hypertension in the adult population has been steadily increasing. There are many hypertensive patients in Korea and most of them take life-long medication for hypertension after the onset of hypertension. THMs for treating hypertension have not yet been developed in Korea. Therefore, patients with hypertension rarely take THMs for the treatment of this disease, but many hypertensive patients take THMs to treat other diseases. For these reasons, patients who take hypotensive drugs often also use THMs simultaneously. However, there are no studies on the interactions between, or guidelines for the coadministration of, hypotensive drugs and THMs.

We evaluated the vasorelaxant effects of the traditional herbal prescriptions most commonly used in Korea on the isolated rat aortic rings. Among them, we found that CWBSD has the most pronounced vasorelaxant effect. In this study, we tested the vasorelaxant mechanisms and hypotensive effect of CWBSD and the vasorelaxant effect and hypotensive effect of combination treatment with CWBSD and amlodipine, which is the most common treatment for hypertension in Korea.

In the present study, CWBSD showed a vasorelaxant effect on isolated rat aortic rings precontracted with PE or KCl and a hypotensive effect on SHRs. In addition, it increased the relaxation of rat aortic rings upon the addition of amlodipine, and coadministration of CWBSD and amlodipine lowered SBP in SHRs more than amlodipine alone.

One of the most important mechanisms for regulating blood pressure is the calcium channel; therefore calcium channel blockers such as amlodipine are the most commonly used agents for hypertension. Vasodilation in vascular smooth muscle is mainly regulated by two different Ca^2+^ influx pathways including ROCCs and VDCCs. These channels can be activated by PE on ROCCs and KCl on VDCCs [24]. In the present study, CWBSD significantly relaxed isolated rat aortic rings precontracted with PE or KCl in a concentration-dependent manner. In addition, CWBSD (600 *μ*g/ml) preincubation for 10 min significantly inhibited the contraction induced by extracellular Ca^2+^ in rat aortic rings precontracted by PE (1 *μ*M) or KCl (60 mM) in Ca^2+^-free KH buffer. These results suggest that the vasorelaxant effects of CWBSD are related to inhibition of the influx of extracellular Ca^2+^ through ROCCs or VDCCs.

CWBSD, one of the most widely used traditional herbal prescriptions in Korea, has been used to treat anxiety disorders such as fright palpitations and fearful throbbing, insomnia, mental fatigue, nocturnal emissions, forgetfulness, dry stool, and stomatitis [25, 26]. This prescription consists of a mixture of herbal medicines, including Liriopis seu Ophiopogonis Tuber, Schisandrae Fructus, and Salviae Miltiorrhizae Radix, which are helpful in treating hypertension or vascular problems. The ethanol extract of* Liriope platyphylla* roots improved vascular dysfunction in the aortic rings of SHR by upregulating antioxidant conditions and downregulating potassium ion (K^+^) and aldosterone concentrations [27]. Gomisin A from* Schisandra chinensis* decreased vascular contraction by inhibiting the RhoA/Rho-kinase signaling pathways [28]. Lithospermic acid B isolated from Salviae Miltiorrhizae Radix showed endothelium-dependent vasodilation in the rat aorta [29]. Puerariae Radix and Salviae Miltiorrhizae Radix decoctions induced vasodilation in the porcine coronary artery through blocking L-type calcium channels and opening K_IR_ channels [30]. These herbal medicines or active components could be responsible for the vasorelaxant and hypotensive effects of CWBSD.

Amlodipine, widely used for the treatment of hypertension, is the clear market leader in Korea. Many antihypertensive medications, such as Norvasc, Anydipine, and Skad, consist of amlodipine. Korean Statistical Information Service (KOSIS) has reported that Norvasc was one of the Top 10 Imported Drug Products of Korea in 2016, with a value of 33 million dollars [31]. Most patients with hypertension in Korea take hypotensive agents containing amlodipine and many of these patients take several kinds of THMs simultaneously to treat other diseases. In the present study, the combination treatment of amlodipine and CWBSD significantly increased vasorelaxation of PE-precontracted rat aortic rings compared to the amlodipine-only treatment. In addition, coadministration of amlodipine and CWBSD also significantly lowered the SBP of SHRs compared to amlodipine alone. These findings suggest that CWBSD does not interfere with the blood-pressure-lowering effect of amlodipine, and it could aid the hypotensive effect of amlodipine through a vasorelaxant effect.

These results suggested that CWBSD could be a useful herbal prescription to treat or prevent hypertension and the hypotensive and vasorelaxant effects of CWBSD were related to attenuation of the influx of extracellular Ca^2+^ through ROCCs or VDCCs. Hence, CWBSD, which has been used for anxiety disorders, is thought to be promising as a traditional herbal prescription for treating hypertension and we recommend establishing guidelines for the use of herbal medicines for treating hypertension in conjunction with antihypertensive drugs including amlodipine.

## 5. Conclusions

The aim of this study was to investigate the vasorelaxant and hypotensive activities of CWBSD and a combination of CWBSD and amlodipine in isolated rat aortic rings and SHRs. CWBSD showed concentration-dependent vasorelaxant activity in endothelium-intact aortic rings precontracted with PE or KCl and lowered the SBP of SHRs. In addition, it increased the relaxation of isolated rat aortic rings induced by amlodipine, and coadministration of amlodipine and CWBSD significantly lowered the SBP of SHRs compared to amlodipine alone. CWBSD relaxed the aortic rings by inhibiting the entry of extracellular Ca^2+^ via ROCCs and VDCCs. These results could be used as basic data for the development of herbal prescriptions for treating hypertension. In addition, further studies on the mechanisms of action and effective dose of CWBSD, and the combination of CWBSD and amlodipine, may provide evidence for the safe administration of CWBSD in patients with hypertension.

## Figures and Tables

**Table 1:** The components of Cheonwangbosimdan.

Herbal name	Scientific name	Part	Collection region	Amount (g)
Rehmanniae Radix	*Rehmannia glutinosa *(Gaertn.) DC. (Plantaginaceae)	Root	Andong, Korea	15.0
Coptidis Rhizoma	*Coptis japonica *(Thunb.) Makino (Ranunculaceae)	Rhizome	China	7.5
Acori Graminei Rhizoma	*Acorus gramineus *Aiton (Acoraceae)	Rhizome	Jeju, Korea	3.75
Ginseng Radix	*Panax ginseng *C.A. Mey. (Araliaceae)	Root	Yeongju, Korea	1.875
Angelicae Gigantis Radix	*Angelica gigas *Nakai (Apiaceae)	Root	Bonghwa, Korea	1.875
Schisandrae Fructus	*Schisandra chinensis *(Turcz.) Baill. (Schisandraceae)	Fruit	Samcheok, Korea	1.875
Asparagi Tuber	*Asparagus cochinchinensis *(Lour.) Merr. (Asparagaceae)	Root	China	1.875
Liriopis seu Ophiopogonis Tuber	*Liriope muscari* (Decne.) L.H. Bailey (syn: *Liriope platyphylla* F.T. Wang & Tang, Asparagaceae)	Root	Miryang, Korea	1.875
Thujae Semen	*Platycladus orientalis* (L.) Franco (syn: *Thuja orientalis* L., Cupressaceae)	Seed	China	1.875
Zizyphi Semen	*Ziziphus jujuba *Mill. (Rhamnaceae)	Seed	China	1.875
Scrophulariae Radix	*Scrophularia buergeriana *Miq. (Scrophulariaceae)	Root	Uiseong, Korea	1.875
Poria Sclerotium	*Wolfiporia extensa* (Peck.) Ginns (Syn: *Poria cocos* (Schwein.) F.A. Wolf, Polyporaceae)	Sclerotium	Pyeongchang, Korea	1.875
Salviae Miltiorrhizae Radix	*Salvia miltiorrhiza *Bunge (Lamiaceae)	Root	China	1.875
Platycodonis Radix	*Platycodon grandiflorus *(Jacq.) A.DC (Campanulaceae)	Root	Muju, Korea	1.875
Polygalae Radix	*Polygala tenuifolia *Willd. (Polygalaceae)	Root	China	1.875
**Total amount**				**48.75**

**Table 2:** Effects of CWBSD on blood pressure in SHRs.

	Systolic blood pressure (mmHg)				
	Time (hour)				
CWBSD (mg/kg)	0 (control)	0.5	1	2	4

247.6	201.3 ± 1.7	203.7 ± 8.0	197.8 ± 12.1	193.2 ± 1.9^*∗*^	207.7 ± 14.4
1,238	207.2 ± 3.8	182.0 ± 8.2^*∗*^	218.7 ± 4.3	219.7 ± 4.8	198.2 ± 3.4
2,476	211.9 ± 2.3	222.4 ± 6.4	172.7 ± 11.2^*∗*^	194.7 ± 8.1	192.5 ± 6.8^*∗*^

Values are expressed as mean ± SEM (n = 5–8). ^*∗*^*p* < 0.05 vs. control.

**Table 3:** Hypotensive effects of coadministration of amlodipine and CWBSD in SHRs.

	Systolic blood pressure (mmHg)				
	Time (hour)				
dose (mg/kg)	0	0.5	1	2	4

Amlodipine (5 mg/kg)	218.8 ± 6.2	199.4 ± 6.6	199.9 ± 15.1	210.7 ± 16.8	168.6 ± 1.5^##^
Amlodipine (5 mg/kg) + CWBSD (2,476 mg/kg)	204.5 ± 12.0	194.6 ± 18.4	215.5 ± 1.4	182.7 ± 12.7	138.2 ± 2.8^##,*∗∗*^

Values are expressed as mean ± SEM (n = 5). ^##^*p* < 0.01 vs. 0 h group. ^*∗∗*^*p* < 0.01 vs. amlodipine group.

## Data Availability

The data used to support the findings of this study are available from the corresponding author upon request.
